# Escape From Treatment; the Different Faces of Leukemic Stem Cells and Therapy Resistance in Acute Myeloid Leukemia

**DOI:** 10.3389/fonc.2021.659253

**Published:** 2021-05-03

**Authors:** Noortje van Gils, Fedor Denkers, Linda Smit

**Affiliations:** Department of Hematology, Amsterdam UMC, location VUmc, Cancer Center Amsterdam, Amsterdam, Netherlands

**Keywords:** therapy resistance, acute myeloid leukemia, leukemic stem cells, minimal residual disease, plasticity

## Abstract

Standard induction chemotherapy, consisting of an anthracycline and cytarabine, has been the first-line therapy for many years to treat acute myeloid leukemia (AML). Although this treatment induces complete remissions in the majority of patients, many face a relapse (adaptive resistance) or have refractory disease (primary resistance). Moreover, older patients are often unfit for cytotoxic-based treatment. AML relapse is due to the survival of therapy-resistant leukemia cells (minimal residual disease, MRD). Leukemia cells with stem cell features, named leukemic stem cells (LSCs), residing within MRD are thought to be at the origin of relapse initiation. It is increasingly recognized that leukemia “persisters” are caused by intra-leukemic heterogeneity and non-genetic factors leading to plasticity in therapy response. The BCL2 inhibitor venetoclax, combined with hypomethylating agents or low dose cytarabine, represents an important new therapy especially for older AML patients. However, often there is also a small population of AML cells refractory to venetoclax treatment. As AML MRD reflects the sum of therapy resistance mechanisms, the different faces of treatment “persisters” and LSCs might be exploited to reach an optimal therapy response and prevent the initiation of relapse. Here, we describe the different epigenetic, transcriptional, and metabolic states of therapy sensitive and resistant AML (stem) cell populations and LSCs, how these cell states are influenced by the microenvironment and affect treatment outcome of AML. Moreover, we discuss potential strategies to target dynamic treatment resistance and LSCs.

## Introduction

**Figure 1:**
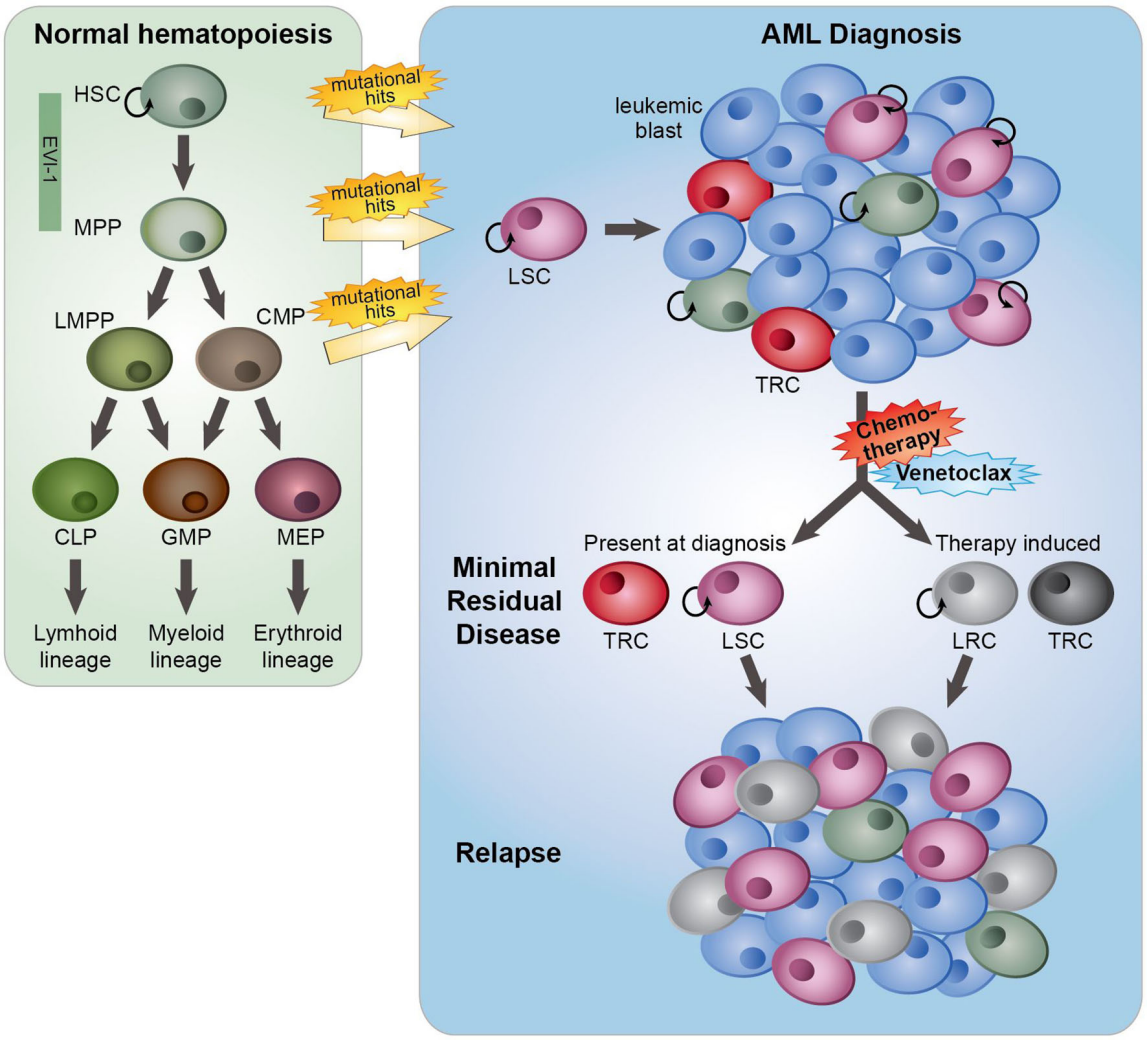
The role of minimal residual disease, therapy resistance and LSCs in AML relapse development. In normal hematopoiesis (green box), quiescent hematopoietic stem cells (HSCs) with self-renewal capacity give rise to multipotent progenitors (MPPs), which can differentiate towards lymphoid primed multipotent progenitors (LMPPs), common myeloid progenitors (CMPs), common lymphoid progenitors (CLPs), granulocyte-macrophage progenitors (GMPs) and megakaryocyte erythroid progenitors (MEP). These lineage-committed progenitors can produce terminally differentiated lymphoid, myeloid and erythroid blood cells. AML originates from the transformation of normal HSCs, MPPs or more committed progenitors, developing in leukemic stem cells (LSCs) that subsequently can give rise to full-blown leukemia. AML initiated from HSC and MPP highly express the transcription factor EVI-1. At AML diagnosis (blue box), a heterogeneous leukemia cell population with a variety of sensitivities to therapy exists. Moreover, LSCs and normal hematopoietic (stem) cells, responsible for reconstituting the normal healthy blood cells after therapy, co-reside in the patient’s bone marrow. While treatment with standard induction chemotherapy results in complete remission in the majority of AML patients, a population of (chemo)therapy-resistant cells (TRCs) (minimal residual disease) constituting AML cells with leukemia-initiating potential survive the treatment. LSCs with leukemia-initiating potential within MRD could initiate sooner or later a relapse. Instead of (chemo)therapeutic selection of pre-existing subpopulations of LSCs, AML cells might adaptively obtain a leukemia re-initiating cell (LRC)-phenotype upon exposure to treatment.

The major problem with cancer treatment is that many patients obtain impressive remissions after a wide variety of treatments yet retain residual tumor cells after the initial therapy, which can develop into recurrence or metastasis. Intratumor heterogeneity in relation to therapy response is the key factor contributing to this treatment failure. For several decades, initial therapy for AML remained unchanged and typically consisted of repetitive courses of intensive combination chemotherapy with anthracyclines and cytarabine, the so-called “7 + 3” standard regimen, aiming at achieving complete remission (CR, <5% of leukemic cells). In general, and dependent on several risk factors, for patients under 60 years the 5-year overall survival (OS) rate after this treatment is 40–50%, while for patients older than 60 years the OS rate is only 15–20%. This poor OS rate in the elderly is partly explained by a higher proportion of patients with an unfavorable disease biology and an inability to tolerate intensive chemotherapy (1). The poor treatment outcome of AML is in part of the patients due to refractoriness to chemotherapy at diagnosis but in the major part caused by relapse originating from a small subpopulation of therapy-resistant leukemia cells (minimal residual disease, MRD) (2, 3) (**Figure 1**). Relapses can occur after months or even years. One of the causes of resistance to anthracyclines is the altered function of the efflux pumps (4), while the efficacy of cytarabine is significantly reduced by enzymatic degradation (5). MRD is the sum of resistance mechanisms to initial therapy, and MRD load is prognostic for OS and relapse-free survival of AML patients (2, 3), indicating that therapeutic targeting of MRD may delay or prevent a relapse, but may also improve the chance of a more successful stem cell transplantation. Leukemia cells with stem cell features (“leukemic stem cells”, LSCs) residing within MRD are thought to be responsible for re-initiation of the tumor (6, 7) (**Figure 1**). Changes in the chromatin and epigenetic landscape, facilitating transcriptional changes, are part of non-genetic therapy resistance, and the presence of a LSC gene expression signature in AML predicts the risk of developing a relapse (8, 9). Also, a LSC expression signature mainly consisting of genes that are epigenetically regulated showed an association with response to chemotherapy (10). Notably, during chemotherapy treatment the frequency as well as the phenotype of LSCs changed, indicating that the treatment itself affects the appearance of relapse-initiating cells (11, 12) (**Figure 1**).

Venetoclax-based therapy could induce responses in approximately 70% of untreated older AML patients (13, 14). However, upfront resistance to venetoclax as well as relapse following CR was appearing (14–17). Recently, it was shown that monocytic AML is less sensitive to venetoclax than immature AML due to the metabolic properties of the monocytic AML cells (18).

**Figure 2:**
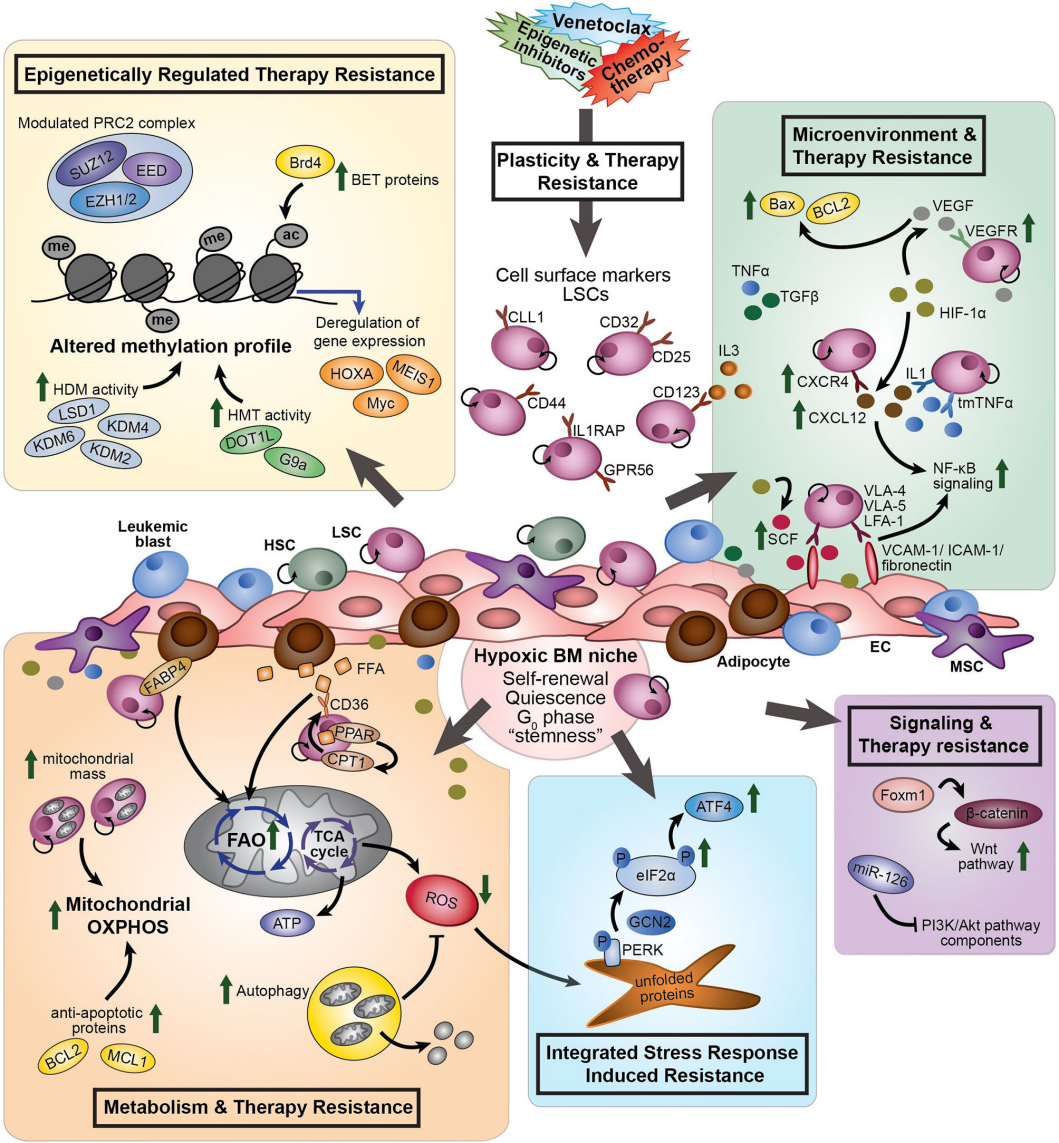
The different processes involved in therapy resistance in AML (stem) cells. *Plasticity and therapy resistance*: Within the hypoxic BM niche, persistent AML (stem) cells might exist prior to drug treatment or might become resistant and obtain leukemia re-initiating potential upon exposure to a treatment, such as chemotherapy or venetoclax. The transcriptional signature of AML LSCs is associated with “stemness” and leukemia-initiating capacity. LSCs aberrantly express lymphoid and myeloid antigens, including CD123, CCL1, CD25, CD32, CD44, GPR56 and IL1RAP. These cell surface markers differ within and between individual AML patients and can change during the course of the disease. Different processes are involved to induce therapy resistance: *Epigenetically regulated therapy resistance* (yellow box): LSCs and therapy-resistant AML cells show modulated expression of components of the PRC2 complex (i.e. EZH1/2), upregulation of BET proteins (*i.e.* Brd4), and altered methylation profile caused by enhanced HDM activity (i.e. LSD1, KDM2, KDM4, and KDM6) and HMT activity (*i.e.* DOT1L and G9a). These differential epigenetic processes induce transcriptional deregulation of genes, like MEIS1, Myc and HOXA. *Microenvironment and therapy resistance* (green box): In response to hypoxia, HIF-1α signaling promotes expression of VEGF, CXCR4, and SCF. CXCR4 on AML cells interact with CXCL12, increasing stromal protective effects. VEGF expressing ECs protect VEGFR3-expressing AML cells from chemotherapy-induced apoptosis, due to increased BCL2/Bax ratios. LSCs express VLA-4, VLA-5 and LFA-1 on their cell surface, which interact with the stromal ligands VCAM-1, ICAM-1 and fibronectin to support attachment to stromal cells, promoting NF-*κ*B signaling. SCF enhances anti-apoptotic and proliferative effects of fibronectin expressed on AML cells. Pro-inflammatory cytokines, including TNFα, influence cell adhesion, promoting LSC survival and chemotherapy resistance through modulation of NF-*κ*B signaling. Several members of the TGF*β* family suppress proliferation of AML cells and enhance chemotherapy resistance. *Metabolism and therapy resistance* (orange box): AML LSCs often lack the ability to enhance glycolysis and therefore switch from anaerobic glycolysis to mitochondria-mediated OXPHOS to generate ATP. Therapy-resistant AML cells show increased mitochondrial mass and high OXPHOS. In addition to glucose, FFA can by metabolized to drive the TCA cycle and OXPHOS. Adipocytes, the major MSC present in the BM, support survival of AML cells by stimulating FAO and OXPHOS through fatty acid transfer. Part of the LSC population expresses the fatty acid transporter CD36 to control uptake of FFA. CPT1, regulated by PPAR, controls FAO translocation by conjugating FFA with carnitine for translocation into the mitochondrial matrix. FABP4 is involved in the interaction of adipocytes with LSCs. Furthermore, LSCs are able to reduce ROS production generated by mitochondria in response to hypoxia, by activation of autophagy. Inhibition of BCL2 by venetoclax efficiently reduces LSC survival by impairing homeostasis and inhibiting OXPHOS. *Integrated Stress Response induced resistance* (blue box): In response to stress stimuli, such as ROS, the PERK-eIF2*α* ISR pathway is activated in LSCs. eIF2*α* is phosphorylated by GCN2 or PERK, reducing global protein synthesis while allowing translation of specific genes, including ATF4. Increased activity of the ISR pathway protects LSCs to enable restoration of homeostasis favoring survival. *Signaling and therapy resistance* (purple box): Upregulation of FOXM1 activates the Wnt/*β*-catenin signaling pathway by direct binding to *β*-catenin, inhibiting its degradation, preserving LSC quiescence and promoting LSC self-renewal. Overexpression of miR-126 repress multiple components of the PI3K/Akt pathway, resulting in the proliferation of LSCs, delayed G_0_ exit of LSCs and enhances resistance to combination chemotherapy. AML, acute myeloid leukemia; ac, acetyl group; ATF4, activating transcription factor 4; ATP, adenosine tri-phosphate; BET, bromodomain and extra-terminal motif; Brd4, bromodomain-containing 4; BM, bone marrow; CCL1, C-type lectin-like molecule 1; CPT1, carnitine O-palmitoyltransferase 1; CXCR4, CXC chemokine receptor-type 4; CXCL12, CXC motif chemokine ligand 12; eIF2*α*, eukaryotic initiation factor 2*α*; EC, endothelial cells; EED, embryonic ectoderm development; EZH1/2, zeste homolog 1 or 2; FAO, fatty acid oxidation; FABP4, fatty acid binding protein 4; FFA, free fatty acids; GCN2, general control non-derepressible 2; HDM, histone demethylase; HIF-1*α*, hypoxia-inducing factor 1*α*; HMT, histone methyl transferase; HSC, hematopoietic stem cell; ICAM-1, intracellular adhesion molecule 1; IL3, interleukin 3; ISR, integrated stress response; KDM, histone lysine demethylase; LFA-1, lymphocyte function-associated antigen 1; LSD1, lysine-specific histone demethylase 1; LSCs, leukemic stem cells; me, methyl group; MSC, mesenchymal stromal cells; NF-*κ*B, nuclear factor *κ*B; P, phosphorylation; PPAR, peroxisome proliferator-activated receptor; PERK, protein kinase RNA-like ER kinase; PI3K, phosphatidylinositol 3-kinase; PRC2, polycomb complex 2; ROS, reactive oxygen species; SCF, stem cell factor; SUZ12, suppressor of zeste 12; TCA, tricarboxylic acid cycle; TGF*β*, transforming growth factor *β*; tmTNFα, transmembrane tumor necrosis factor α; VCAM-1, vascular cell adhesion molecule 1; VEGF, vascular endothelial growth factor; VEGFR, vascular endothelial growth factor receptor; VLA-4, very late antigen-4; VLA-5, very late antigen-5.

The biggest challenge in the treatment of AML is relapse and refractoriness caused by persistent AML cells that survive the initial treatment. The term “drug tolerant persisters” is frequently used to describe cancer cells with non-mutational mechanisms of drug resistance. Persistent leukemia cells might exist prior to drug treatment; however, they might also become resistant upon exposure to a treatment (**Figure 1**). As shown decades before with bacteria, persistent tumor cells can resume their drug sensitivity upon drug removal (19). Leukemia “persisters” are characterized by their quiescence state, different energy consumption, adaptation to the bone marrow (BM) microenvironment, changing identity, and phenotypic plasticity. Mechanisms that cause their persistence include a variety of epigenetic, transcriptional, and metabolic processes that often co-exist (**Figure 2**). Clinical targeting of persistent AML cells (MRD) will increase efficacy of treatment and finally the survival of patients. For the development of successful therapeutic strategies targeting AML MRD, it will be crucial to understand the mechanisms that drive this persistence.

## The Heterogeneity and Plasticity of LSCs and Minimal Residual Disease

AML has a hierarchical cellular organization, with a small fraction of self-renewing LSCs at the apex of the hierarchy. LSCs are defined as cells that can self-renew, which was experimentally shown by the capacity of re-initiation of leukemia when (serial) transplanted into immunodeficient mice. Moreover, LSCs can differentiate into non-LSC blasts (20, 21). The identity of LSCs is influenced by clonal genetic evolution, epigenetic alterations, their metabolic state, and their microenvironment, finally resulting in intra- and interpatient heterogeneity in their response to therapy (8, 22–24) (**Figure 2**). In AML, LSCs have been described as a heterogeneous and relatively rare cell population that could be isolated from the leukemic bulk population by flow cytometry based on expression of a set of specific cell surface markers, including CD34+CD38− (20, 21). In general, CD34+CD38− AML cell populations display a higher leukemia-initiating cell frequency than CD34+CD38+ AML cell populations (8, 22). Interestingly, in 80% of CD34-positive AML cases, at least two distinct LSC populations were identified, a CD34+CD38− fraction resembling normal lymphoid primed multipotent progenitors (LMPP-like LSCs) and a CD34+CD38+ fraction resembling granulocyte-macrophage progenitors (GMP-like LSCs) which have been derived from the LMPP-like LSC population. In almost 15% of CD34-positive AML cases, there is a dominant population of LSCs that resembles multipotent progenitors (MPP-like LSCs) (25) (**Figure 1**). Understanding the biological properties of AML LSCs, particularly their similarities and differences from normal CD34+CD38− hematopoietic stem cells (HSCs) and their heterogeneity and plasticity in individual patients is important for the development of therapies that can specifically eradicate these cells during the course of disease.

In the past, the identification of cell surface markers differentially expressed between LSCs and HSCs has been intensively studied, but thus far no unique marker universally expressed on CD34+CD38− LSCs across AML patients but not on normal HSCs has been discovered. This is mainly due to the intra- and interpatient heterogeneity of AML. Many lymphoid and myeloid antigens are aberrantly expressed in AML, which give rise to complex leukemia-associated (immune)phenotypes (LAIPs) that are highly heterogeneous and differ between the individual AML patient (26). Expression of these LAIPs can also change during the course of the disease (27). Despite this heterogeneity, it was shown that specific cell surface markers are increased on LSCs compared to HSCs or progenitors in part of the AML patients, including CD123 (28), C-type lectin-like molecule-1 (29), CD25 (30), CD32 (30), CD44 (31), IL1RAP (32), and GPR56 (33) (**Figure 2**).

During the development of AML, genetic aberrancies induce epigenetic changes, leading to increased epigenetic plasticity in leukemic cells. While it was shown that genetic mutation-driven, unique epigenetic profiles in leukemic cells could be developed (34), LSCs demonstrated to share an epigenetic signature that is mostly independent of genetic mutations (10). Accordingly, transcriptional profiling of AML LSCs revealed a molecular signature that is associated with leukemia “stemness” and leukemia-initiating capacity and that is highly correlative with AML prognosis (8, 9), indicating that the presence of a transcriptional “stemness” profile affects response to treatment. Moreover, reactivation of a self-renewal-associated transcriptional signature was shown to be an important characteristic of the transformation of normal progenitors into LSCs (35). Together, these results implicated that there is high degree of plasticity in imposing “stemness” on leukemic cells, and showed the importance of stem cell features for the response to chemotherapy of AML cells.

Recently, several studies challenged the idea that LSCs are less sensitive to chemotherapy than non-LSCs. These studies showed that LSCs are not selectively resistant to chemotherapy (12, 36, 37), and that the identity of the LSC is transient and dynamic during the AML disease course (11, 12). Enhanced LSC frequencies and phenotype diversity were observed at relapse as compared to diagnosis, suggesting that current AML therapeutic regimens promote dramatic changes in the LSC compartment. Instead of chemotherapeutic selection of pre-existing subpopulations of drug-resistant AML LSCs, AML cells adaptively acquired a leukemia re-initiating cell (LRC)-specific signature, implying that LRCs are developed in response to chemotherapy (12). Interestingly, as these LRCs express a LRC-specific gene signature, it might be very useful to measure LRC markers after initial therapy for the early detection of relapse initiation. Thus, LRCs were described to emerge after chemotherapy treatment and differed from LSCs at diagnosis (12) (**Figure 1**), indicating that it is most relevant to study the relapse-initiating cells after the initial treatment.

## The Quiescent and Therapy Resistant State of LSCs

Cell-intrinsic, epigenetic, and transcriptional reprogramming leading to reduced cell proliferation is associated with reduced sensitivity to treatment and an increased tumor-initiating potential (**Figure 2** **)**. This reduced proliferative cell state might exist before therapy but can also be acquired as a result of treatment. For example, persistent cancer cells derived from human glioblastoma patients entered a slow-proliferation state following treatment with the tyrosine kinase inhibitor dasatinib (38). In patient-derived xenografts (PDX) of primary acute lymphoblastic leukemia (ALL), a rare dormant subpopulation of ALL cells resembling relapse-inducing cells were treatment resistant and contained “stemness” properties (39). While most of the clonogenic AML cells in AML patient samples were actively cycling, a small number of AML progenitors were quiescent. Furthermore, leukemia cells capable of engrafting in NOD/SCID mice showed to be quiescent and in the G_0_ of the cell cycle prior to transplantation. After serial transplantation a rare quiescent long-term human SCID leukemia-initiating cell population with extensive self-renewal capacity and extremely low proliferation rate was identified, suggesting that only a small proportion of the LSC pool has extensive self-renewal potential and drives progression to AML (40).

CD34+CD38− LSCs in AML reside in the endosteal region of mouse bone marrow, wherein they are primarily quiescent and protected from cytarabine-induced apoptosis (7). These AML LSCs could be activated to enter the cell cycle and become sensitized to cytarabine by administration of exogenous granulocyte colony-stimulating factor (41). Also, interleukin (IL)-3, a critical cytokine involved in myeloid differentiation and the ligand of CD123, could enhance the proliferation rate of AML blasts. CD123 is involved in the potential of LSCs to engraft in NOD/SCID mice, as blocking CD123 with the monoclonal antibody 7G3, in the absence of exogenous human cytokines, inhibited the engraftment and growth of AML CD34+CD38− cells (28). Together, these results indicated that CD34+CD38− LSCs are more quiescent than bulk AML cells, but can still respond to hematopoietic growth factors thereby entering the cell cycle.

MicroRNA (miR)-126 was identified as a critical regulator of LSC quiescence (**Table 1**). Overexpression of miR-126 expanded primitive CD34-positive cells, delayed the G_0_ exit of CD34+CD38− LSCs, while the G_0_ status of CD34+CD38+ and CD34-negative cell populations remained unaffected. Moreover, miR-126 overexpression enhanced resistance to the combination treatment of daunorubicin and cytarabine by preserving LSCs in a quiescent state (42). Thus, miR-126 can keep CD34+CD38− AML cells in a more primitive state by increasing the proportion of quiescent CD34+CD38− cells, thereby decreasing the overall proliferative output and differentiation of AML blasts. miR-126 is highly expressed in LSCs as compared to leukemic progenitors, and high miR-126 expression in AML is associated with poor OS and a higher chance of relapse. Targeting miR-126 in LSCs reduced their clonogenic capacity, in the absence of an inhibitory effect on normal BM cells (43). Multiple components of the phosphatidylinositol 3-kinase (PI3K)/Akt pathway are repressed by miR-126 (42), consistent with previous mouse studies demonstrating that the PI3K/Akt pathway plays a key role in governing quiescence and regulating HSC and LSC self-renewal (44, 45) (**Table 1**). Although most known self-renewal regulators have comparable functions in HSCs and LSCs, regulation of the cell cycle by miR-126 is opposite. Reduced miR-126 levels resulted in HSC expansion, while the maintenance of LSC was impaired (42). Accordingly, expression of a constitutively active form of Akt in HSCs (45) or loss of the negative regulator PTEN (44) resulted in HSC exhaustion and LSC expansion, suggesting that targeting the PI3K/Akt signaling pathway, like targeting miR-126, will differentially affect quiescence and self-renewal in HSCs and LSCs.

**Table 1:** Therapeutic targets and drugs to overcome therapy resistance mechanisms in AML (stem) cells.

AML (stem) cell therapy resistance mechanism	Target protein or process	Drug	Preclinical studies	Clinical trial for AML	References
Quiescence	miR-126	N/A	Reduction of clonogenic capacity of LSCs in the absence of an inhibitory effect on normal BM cells.	N/A	(42, 43)
	PI3K/Akt; PTEN	Rapamycin	Depletion of leukemia-initiating cells and restoration of normal HSC function.	- Phase I: rapamycin + decitabine in r/r AML - Phase I: rapamycin + chemotherapy in newly diagnosed AML, r/r AML and secondary AML - Phase II: rapamycin in r/r AML	(44, 45)
	FOXM1	Thiostrepton	Reduction of self-renewal capacity of LSCs in MLL-rearranged AML, synergistic effects with chemotherapy on induction of apoptosis in LSCs, and prolonged survival *in vivo*.	N/A	(46)
Epigenetically driven drug resistance	LSD1	Iadademstat (ORY-1001)	LSD1 target gene specific increase of H3K4me2, induction of AML blast differentiation and reduction of LSC self-renewal capacity, while sparing normal CD34+ cells.	Phase I/IIa in r/r AML (non-M3) and r/r MLL-rearranged AML	(47–49)
		TCP	Induction of differentiation of AML blasts and inhibition of AML cell growth.	Phase I/II: ATRA + TCP in r/r AML (non-M3)	(50, 51)
		GSK-LSD1	Myeloid differentiation in MLL-rearranged AML cells, causing global gains in chromatin accessibility, with an enrichment of PU.1 and C/EBP*α* at these open sites.	N/A	(52)
	EZH2 and/or EZH1	DZNEP	Reduced EZH2 and H3K27me3 levels, resulting in reduced CD34+CD38- LSC numbers. In combination with panabinostat, synergistic induction of apoptosis in AML cells, while sparing normal CD34-positive BM progenitor cells.	N/A	(53, 54)
		OR-S1	Reduction of LSC numbers, impaired AML progression and prolonged survival *in vivo*. Priming AML cells for chemotherapy-induced cell death.	N/A	(55)
		Valemetostat (DS-3201)	Recruitment of quiescent AML LSCs into cell cycle.	Phase I in r/r AML	(56)
	G9a	CM-272	Activation of interferon response, inhibiting proliferation and promoting apoptosis. Prolongation of OS in AML xenogeneic mouse models.	N/A	(57)
		Pinometostat (EPZ5676)	Tumor growth suppression, reduced colony-forming capacity, and terminal differentiation in DNMT3A-mutated AML cells.	Phase I in MLL-rearranged AML	(58, 59)
	BET proteins	JQ1	Anti-leukemic effects accompanied by terminal differentiation and elimination of LSCs. Reduction of BCL2 and c-myc levels, inducing apoptosis in NPM1c+ with or without FLT3-ITD or MLL-rearranged AML. In combination with panabinostat, synergistic induction of apoptosis in AML, while sparing normal CD34-positive BM progenitors.	N/A	(60, 61)
		Birabresib (OTX015)	Inhibition of cell growth, cell cycle arrest and apoptosis in AML cells.	Phase I in r/r AML	(62)
		Molibresib (GSK525762)	Downregulation of BCL2, c-myc and IRF8, reduction in clonal growth and induction of apoptosis in AML cells, and survival advantage *in vivo*.	Phase I/II in r/r AML and secondary AML	(63)
Hypoxia and metabolism	BCL2	Venetoclax	Inhibition of OXPHOS and impairing energy homeostasis, upregulation of myeloid differentiation genes, and downregulation of cell cycle and proliferation genes.	Phase Ib and phase III: venetoclax + azacitidine in elderly AML Phase Ib: venetoclax + azacitidine or decitabine in elderly AML Phase II: venetoclax in r/r AML Phase III: venetoclax + cytarabine in newly diagnosed AML ineligible for intensive chemotherapy	(13–17, 64, 65)
	MCL-1	(−)BI97D6	Induction of mitochondrial apoptosis in AML, due to disrupted MCL-1/BIM and BCL2/Bax interactions, while sparing normal hematopoietic stem/progenitor cells.	N/A	(66)
		VU661013 + venetoclax	Destabilization of BIM/MCL1 and induction of apoptosis in AML. Synergistic reduction in tumor burden after combination therapy.	N/A	(67)
		AZD5991	Induction of apoptosis in AML by activation of Bak-dependent mitochondrial apoptosis, and anti-tumor activity.	Phase I/II: AZD5991 monotherapy or in combination with venetoclax in r/r AML	(68)
		AMG176	Rapid induction of apoptosis in AML, growth inhibition of human AML *in vivo*.	Phase I: AMG176 + azaciditine in r/r AML	(69)
		MIK665 (S64315)	Induction of AML cell death, induction of antitumor responses after combination treatment with a BCL2 inhibitor.	Phase I: S64315 in r/r AML (non-M3) Phase I: VOB560 (BCL2 inhibitor) + S64315 in r/r AML	(70)
	HIF-1*α*	Echinomycin	Inhibition of colony formation, induction of apoptosis of CD34+CD38- AML cells. Elimination of leukemia initiating cells and reduction in human leukemic burden.	N/A	(71)
		TH-302	Hypoxia-dependent apoptosis in AML cells, by reducing HIF-1*α* expression, decreasing proliferation, inducing a cell-cycle arrest, and enhancing double-stranded DNA breaks. Prolongation of residual disease after chemotherapy treatment *in vivo*.	N/A	(72, 73)
Bone marrow micro-environment	CXCR4	Plerixafor (AMD3100)	Mobilization of AML blasts from the BM niche into peripheral circulation, sensitization of leukemic blasts to cytarabine and decreased tumor burden *in vivo*.	Phase I/II: plerixafor + mitoxantrone, etoposide and cytarabine in r/r AML Phase I/II: plerixafor + decitabine in newly diagnosed elderly Phase I/II: plerixafor + fludarabine, idarubicin, cytarabine and G-SCF in r/r AML	(74–76)
		ARV-825	CXCR4 and CD44 downregulation, impairment of CXCL12-directed migration, increased oxidative stress, downregulation of gene signatures associated with stemness, Wnt/*β*-catenin and Myc pathways, and decrease in number of LSCs.	N/A	(77)
	TGF*β*	1D11	Enhanced cytarabine-inducted apoptosis of AML cells in hypoxic conditions. Combination treatment with plerixafor and cytarabine decreased leukemia burden in FLT3-mutated mice.	N/A	(78)
	VEGF-C	VGX-100	Reduction of clonogenic capacity and induction of differentiation of AML blasts, *via* suppression of FOXO3A and inhibition of MAP/ERK proliferative signals.	N/A	(79)
		Bevacizumab	N/A	Phase I: bevacizumab n r/r AML Phase II: bevacizumab + mitoxantrone + cytarabine in r/r AML Phase II: bevacizumab + daunorubicin + cytarabine in newly diagnosed elderly	(80) (81, 82)
Adipocytes	FABP4	BMS309403	Inhibition of AML blast survival, while sparing nonmalignant CD34-positive cells.	N/A	(83)
	FAO	Etomoxir	Disruption of metabolic homeostasis in AML cells, induction of ROS production and ATF4. Inhibition of CPT1a and subsequent sensitization of AML cells to cytarabine. Induction of an energetic shift towards low OXPHOS and increase in anti-leukemia effects of cytarabine.	N/A	(36) (84) (85)
		Avocatin B	Upregulation of ATF4 and synergistic induction of ROS production and apoptosis in AML cells after combination treatment with cytarabine.	N/A	(84)
Stress response	Autophagy	VSP34 inhibitors	Inhibition of autophagy and cell proliferation abolishes acquired FLT3 inhibitor resistance.	N/A	(86)
	PERK/eIF2*α* pathway	Atovaquone	Phosphorylation of eIF2α, enhancing ATF4 protein expression and ATF4-specific target genes, inhibiting OXPHOS, and inducing growth arrest and apoptosis in AML cells.	N/A	(87)
		GSK2606414 + BIX-01294	Synergistic induction of apoptosis in AML cells, while sparing normal HSCs.	N/A	(88)

LSC, leukemic stem cell; HSC, hematopoietic stem cell; BM, bone marrow; r/r AML, relapsed and refractory acute myeloid leukemia; OS, overall survival; ROS, reactive oxygen species; oxidative phosphorylation, OXPHOS, N/A, not applicable.

Another promising therapeutic target to eliminate quiescent AML LSCs is FOXM1 (**Table 1**). *Foxm1* upregulation activated the Wnt/*β*-catenin signaling pathway by direct binding to *β*-catenin and stabilizing *β*-catenin through inhibition of its degradation, thereby preserving LSC quiescence, and promoting LSC self-renewal in MLL-rearranged AML. Targeting FOXM1 inhibited the survival, quiescence, and self-renewal of MLL-AF9-transformed LSCs (46).

The WT1 gene and HCK, a member of the Src family of tyrosine kinases, were identified as being more highly expressed in cell cycle-quiescent primary AML LSCs than in normal HSCs. Moreover, comparison of cell surface markers expressed on LSCs and normal HSCs revealed that CD32 and CD25 are promising therapeutic targets to specifically eradicate LSCs. While CD32 and CD25 are not or less expressed on normal HSCs, these markers are expressed in a large fraction of primary human AML LSCs (52.5% of the AML cases express CD32, CD25, or both on the LSCs), stably located on the LSC cell surface after chemotherapy treatment and present on cell cycle-quiescent AML-initiating cells residing within the endosteal niche (30).

Although several studies suggested that LSCs withstand chemotherapy regimens due to quiescence and dormancy (7, 41), this concept was recently challenged. In a AML PDX mouse model, residual AML cells were neither enriched in immature quiescent cells, nor in LSCs after treatment with cytarabine, suggesting that cytarabine similarly depleted quiescent G_0_ AML cells and proliferating cells (36). LSCs were shown to have a variety of sensitivities to cytarabine, and treatment with cytarabine reduced the frequency of leukemia-initiating cells by inducing an exit from G_0_, increasing proliferation and subsequent depleting part of the LSCs. Transcriptional profiling of cytarabine residual AML cells that contained leukemia-regenerative capacity showed that cytarabine sensitive and -resistant LSCs have a distinct transcriptome (12). Together, these results suggested that part of the LSC pool is not inherently resistant to chemotherapy, and also showed that quiescence or dormancy is not sufficient to protect LSCs from chemotherapy-induced cell death. Moreover, these results suggested that LSCs can acquire genetic and epigenetic alterations and exhibit phenotypic plasticity to adjust to environmental changes, highlighting the dynamic properties of AML LSCs during the course of therapy.

## Epigenetically-Driven Drug Resistance in AML

Epigenetic changes contribute to resistance to chemotherapy and targeted treatments (89–91) (**Figure 2**). Epigenetic mechanisms driving sensitivity to therapy in the individual leukemia cell could be established by genetic aberrations, by signaling from the microenvironment but also by the leukemia cell of origin. Currently, it is increasingly recognized that therapeutic resistance in the absence of a genetic aberrancy is a major cause of recurrence and metastasis in several cancers, including AML (91–94). As epigenetic modifications, such as DNA methylation and post-translational modifications on histone tails, are reversible, these epigenetic marks provide great opportunities to target non-genetic therapy resistance using specific epigenetic inhibitors.

Since LSCs and leukemic blasts share a common set of genetic mutations in most AML cases, functional properties that differ between these two cell compartments are likely driven by epigenetic differences. Indeed, multiple differentially methylated regions were identified between LSC-containing and non-LSC-containing cell populations. In LSCs, these regions were predominantly hypomethylated and largely associated with transcriptional upregulation. The DNA methylation signature differentially present in LSCs compared to leukemic progenitors consisted of 71 genes, which were enriched for HOX genes, and which were associated with a poor prognosis independent of other known risk factors (10). In addition to DNA methylation, histone tail modifications, including acetylation, methylation, and ubiquitination contribute to transcriptional output. The methylation of histones is dynamically regulated, and depending on the position and nature of the methylated residues, can either promote or repress transcription. Histone methyltransferases (HMTs) add methyl groups to specific histone residues, while histone demethylases (HDMs) remove methyl groups from specific residues on histone tails (95). In general, the methylation on histone 3 (H3) lysine-4 (H3K4), H3K36, and H3K79 activate gene expression, whereas methylation on H3K9, H3K27, and H4K20 is associated with transcriptional repression. LSCs in MLL-rearranged leukemia were characterised by high levels of H3K4me3 and low H3K79me2, resulting in aberrant expression of HOX genes and Meis1 (96). The H3K4 lysine specific demethylase 1 (LSD1; KDM1A) is highly expressed in AML, and associated with transcriptional repression (97). LSD1 can demethylate mono- and di-methylated H3K4 and H3K9, but also has a scaffolding activity that facilitates recruitment of histone deacetylases to chromatin sites where transcription factors such as GFI and GFIB are bound (98). Moreover, LSD1 sustains the differentiation block in certain molecular subtypes of AML, particularly in MLL-translocated AML, and is required for the self-renewal potential of LSCs (97, 99). Targeting LSD1 in AML promoted differentiation (100), and compromised the self-renewal capacity of LSCs in (pre)clinical models of AML (**Table 1**). For example, iadademstat (ORY-1001), a covalent and highly specific LSD1 inhibitor, induced a gene-specific increase of H3K4me2, resulting in induction of AML blast differentiation and reduction of LSC self-renewal capacity, while sparing normal CD34-positive cells (47). In phase-I clinical trials, treatment of relapsed/refractory (r/r) AML patients with iadademstat resulted in the induction of differentiation of the leukemic blast cells, and reduction of blast percentages in peripheral blood and BM. In some individual patients there was a relation between response and induction of *CRISP9* (48, 49)*, CD86*, *VCAN*, *S100A12* and *LY96* (48). However, In a phase-I/II clinical trial using the LSD1 inhibitor tranylcypromine (TCP) in combination with all-trans retinoic acid (ATRA) for r/r AML patients, there was an overall response rate of only 20%. Molecular markers associated with response were not identified and a global increase in H3K4me2 upon TCP was only observed in two patients (50). In non-responders to TCP and ATRA combination therapy there was enrichment for expression of genes involved in mTOR signaling and for expression of higher basal histone deacetylase 2 (*HDAC2*) (51). Moreover, treatment of MLL-rearranged AML with the LSD1 inhibitor GSK-LSD1 caused global gains in chromatin accessibility, with a strong enrichment of PU.1 and C/EBP*α* at these open sites. Depletion of PU.1 or C/EBP*α* generated resistance to LSD1 inhibition (52).

Next to LSD1, expression of other HDMs, including the H3K27 demethylase KDM6B, the H3K36 demethylase KDM2B, and the H3K9/36me3 demethylase KDM4A correlated to treatment response. KDM6B is increased in AML as compared to normal BM, and positively correlated with poor survival. Treatment with the KDM6 inhibitor GSK-J4 enhanced the global levels of H3K27me3 and showed synergistic effects with cytarabine. GSK-J4 treatment decreased the expression of cell-cycle related pathways and HOX genes in AML cells (101). KDM2B interacts with active chromatin, is overexpressed in LSCs, and can function as the DNA binding subunit of the polycomb repressive complex 1 (PRC1). Knockdown of KDM2B was shown to impair the self-renewal capacity of LSCs (102). Inhibition of KDM4A by JIB-04 restored the levels of H3K36me3 but also induced sensitivity to chemotherapy (103). Together, these studies indicate crucial roles for several HDMs in non-genetic therapy resistance in AML, and show their potential as therapeutic targets to deplete LSCs and/or MRD.

PRC2 is one of the two classes of polycomb-group (PcG) protein complexes. PRC2 contains histone methyltransferase activity and primarily trimethylates H3K27, leading to silencing of target gene transcription, while PRC1 is able to condense nucleosomes, inducing stable gene silencing. The PcG proteins are required for long term epigenetic silencing and have an important role in maintaining “stemness” (104). The enhancer of zeste homolog 2 (EZH2) is a member of the PRC2 complex, mediating transcriptional silencing through H3K27me2/3 (105). In AML, quiescent LSCs express the highest levels of EZH1 and EZH2 (55). In about 45% of relapsed AML, loss of EZH2 and consequently a reduction in H3K27me3 occurs. This EZH2 loss resulted in resistance toward multiple drugs, including tyrosine kinase inhibitors (90), and is associated with a poor OS (106). EZH2 loss may be mediated by the interaction between cyclin-dependent kinase 1 and heat shock protein 90, that induces EZH2 proteasomal degradation, and drug resistance *via* deregulation of HOX gene expression (90). Loss of EZH2 in AML can also be due to a 7/7q chromosomal deletion, as EZH2 is located on chromosome 7q36.1, or can be caused by splicing dysfunction as a result of mutations in U2AF1 or SRSF2. Genetic aberrancies in the U2AF1 and SRSF2 genes have been shown to decrease EZH2 mRNA levels in about 10-25% of AML patients (107, 108). AML and myelodysplastic syndrome patients with a 7/del7q are largely refractory to chemotherapy and have a particular poor prognosis (109). Also presence of U2AF1 and SRSF2 mutations in AML is associated with adverse outcome (110), which might be explained by loss of EZH2-driven resistance to treatment (90). Interestingly, low H3K27me3 levels in AML samples, potentially as a result of low EZH2, is also a parameter for a poor prognosis (90, 106). However, the role of EZH2 in therapy resistance in AML is complex and may depend on the context. Indeed, a stage-specific and opposite function for EZH2 at the early and late stages of the disease was suggested; EZH2 acted as a tumor suppressor at the stage of AML induction, while it exerted an oncogenic function during leukemia maintenance (111). Moreover, the therapeutic effect of EZH2 inhibition may be dependent on the treatment with which it is combined. The EZH2 inhibitor 3-Deazaneplanocin A (DZNEP) in combination with the HDAC inhibitor panabinostat synergistically induced apoptosis in primary AML cells, while not affecting the survival of normal CD34-positive BM progenitor cells (53). DZNEP reduced EZH2 and H3K27me3 levels, resulting in a reduction in the number of CD34+CD38- LSCs (54) (**Table 1**). Quiescent LSCs are highly dependent on both EZH1 and EZH2, and dual inhibition of EZH1 and EZH2 by OR-S1 primed AML cells for chemotherapy-induced cells death (**Table 1**). Moreover, OR-S1 reduced the number of LSCs, impaired leukemia progression and prolonged survival of AML PDX mice (55). In a phase-I clinical trial, inhibition of EZH1/2 with valemetostat drove quiescent AML LSC into the cell cycle (56). Based on these results, it would be of interest to investigate if EZH1/2 inhibition reduces MRD load and/or LSCs after chemotherapy- and/or venetoclax treatment.

Apart from PRC2, other HMTs can regulate methylation on histone tails, including DOT1L and G9a. G9a catalyzes mono- and di-methylation of H3K9 and induces changes in redox homeostasis (112). G9a was shown to accumulate under hypoxic conditions (113), and as AML LSCs are known to reside in hypoxic BM niches, therapeutic targeting of G9a may efficiently eliminate AML LSCs (**Table 1**). Indeed, loss of G9a impaired AML progression and reduced LSC frequency (114). CM-272, a small molecule simultaneously inhibiting G9a and demethyltransferase (DNMT)-1 activity, inhibited proliferation, promoted apoptosis in AML cells, and prolonged the OS in AML xenogeneic mouse models (57). The HMT DOT1L plays a key role in initiation and maintenance of MLL-rearranged leukemia, because of its role in H3K79 methylation and subsequent upregulation of Meis1 and HOXA (115). Inhibition of DOT1L by EPZ5676 suppressed tumor growth, reduced colony-forming capacity, and induced terminal differentiation in DNMT3A-mutant AML cells (58). In a phase-I study, treatment with EPZ5676 resulted in a significant reduction in H3K79me2 levels, while CR was only achieved in two of the 51 r/r MLL-rearranged AML patients (59), indicating that anti-DOT1L monotherapy is not sufficient to achieve clinical benefit in r/r AML patients.

Other epigenetic modifiers playing a critical role in the maintenance of AML are bromodomain and extra-terminal motif (BET) proteins, which sustain Myc expression to promote aberrant self-renewal (116). BET family proteins, including bromodomain-containing 4 (Brd4), facilitate gene transcription by binding to acetylated lysines in histones and transcription factors. Brd4 was identified as a promising therapeutic target for AML (60, 61, 117) (**Table 1**), and targeting Brd4 using small hairpin RNAs or small molecule inhibitors resulted in a strong anti-leukemia effect, terminal myeloid differentiation and elimination of LSCs (60). Despite these promising preclinical results, monotherapy with BET inhibitors showed limited efficacy and CR was only induced in a few AML patients (62, 63). Resistance to BET inhibitors emerges from LSCs in the absence of new genetic mutations (93), and is acquired through adaptive transcriptional plasticity and the conversion of AML cells to a more immature LSC phenotype. Regulators of enhancer formation were identified as key mediators of the resistant state (92), but also chromatin remodelling, leading to activation of the Wnt signalling pathway, was shown to be involved in BET inhibitor resistance (93, 118). BET inhibitor resistant AML cells use available factors, such as PU.1 and interferon regulatory factor 8 (IRF8), to nucleate the different enhancers, facilitating remodelling of regulatory pathways that rapidly restore expression of survival genes (92, 118). Reversion of the BET inhibitor resistant phenotype was accomplished by targeting the mechanisms whereby AML cells use alternative enhancers. LSD1 inhibition is able to re-sensitize stable BET inhibitor resistant AML cells, by facilitating enhancer switching mediated by PU.1 and IRF8 (92). Although it has yet to be seen whether resistance to other (epigenetic) drugs work *via* similar mechanisms, these results suggest that rather than aiming at reversion of the transcriptional state of resistant cancer cells, it may be more effective to disable the process of enhancer remodeling.

## AML Therapy Sensitivity and the Cell of Origin

Epigenetic states conferred by cell of origin shape the molecular classification across a diverse array of tumor subtypes. The cell of origin of leukemic transformation is also a determinant of therapy sensitivity. Analysis of genetic abnormalities in paired AML samples at diagnosis and relapse indicated that leukemia-initiating cells within MRD can originate from rare LSCs, from a dominant subclone with a HSC phenotype, or from subclones of immunophenotypically committed leukemia cells that retained “stemness” (94). Leukemia initiated from HSC demonstrated to exhibit higher disease penetrance, aggressiveness, and resistance to chemotherapy and have higher expression of the transcription factor Mecom (EVI-1) than leukemia arising from more differentiated progenitor cells (119–122) (**Figure 1**). High expression of EVI-1 is a prognostic factor associated with inferior OS among AML patients harbouring MLL gene rearrangements (123). Moreover, HSC-derived leukemia exhibit decreased apoptotic priming, attenuated p53 transcriptional output, and resistance to LSD1 inhibitors. The expression of EVI-1 modulates the abundance and activity of the p53 protein. Interestingly, EVI-1^high^ AML cells are sensitized to LSD1 inhibition by venetoclax (122), suggesting that immature AML cases, which have their origin in the HSC or MPP, can be sensitized for anti-LSD1-induced apoptosis by therapeutic targeting of BCL2. The level of response to the combination of venetoclax and azacitidine is also related to the differentiation stage of the AML. More immature, primitive AML, likely originating from a more immature cell, such as the HSC or MPP, was more sensitive to venetoclax than differentiated monocytic AML. Venetoclax-resistant monocytic AML had a distinct transcriptomic profile, reduced expression of BCL2, and showed to rely on MCL1 for oxidative phosphorylation (OXPHOS) and survival. Consequently, there was outgrowth of monocytic subpopulations of AML cells after venetoclax treatment at relapse (18). Targeting these venetoclax-resistant monocytic leukemia cells might be accomplished by therapies that are highly efficient in eradicating more differentiated as compared to immature AML.

## Hypoxia, Metabolism, LSCs and Therapy Resistance

Hypoxia, a condition in which the normal tissue oxygen level is reduced, has been identified as a major contribution to resistance to many drugs and an enhanced tumorigenicity of cancer stem cells (124). The transcriptional regulator hypoxia-inducible factor 1 alpha (HIF-1*α*) responds to hypoxia by binding to hypoxia-response elements and by regulating expression of hypoxia-response genes, finally resulting in activation of enzymes involved in DNA repair, cell differentiation and apoptosis (125). In hypoxic tumor cells, the accumulation of chemotherapeutic drugs is reduced, and induction of drug resistance by increased genomic instability, suppression of DNA repair and suppression of a cell cycle arrest can occur (126).

HSCs reside in hypoxic niches in the BM, in where HIF-1α signaling regulates the maintenance of their quiescent and pluripotent state by promoting HIF-1α-driven gene expression, including vascular endothelial growth factor (VEGF), CXC chemokine receptor-type 4 (CXCR4) and stem cell factor (SCF) (127). Since LSCs are well adapted to hypoxic conditions, there is a protective effect of the niche on the persistence of these LSCs, which results in their reduced sensitivity to therapy. Under hypoxic conditions, reactive oxygen species (ROS) are generated by mitochondria. In contrast to HSCs, LSCs are able to reduce generation of ROS and induce the activation of ROS removing pathways such as autophagy, resulting in enhanced survival of LSCs compared to HSCs (128).

Through glycolysis, glucose is metabolized to pyruvate, and in the presence of oxygen pyruvate can be further metabolized to acetyl-CoA, that is oxidized in the tricarboxylic acid (TCA) cycle to drive OXPHOS and the generation of ATP. Although AML LSCs are mainly in a low oxidative and quiescent cell state, they often lack the ability to enhance glycolysis and therefore switch from anaerobic glycolysis to mitochondria-mediated OXPHOS as their major pathway to generate energy, while HSCs rely mainly on anaerobic glycolysis (64). Also, residual AML cells after cytarabine treatment showed an increased mitochondrial mass, and retained active polarized mitochondria, reflecting a high OXPHOS status. Moreover, presence of a high OXPHOS gene expression signature was predictive for a worse treatment outcome in AML patients (36). The deacetylating mitochondrial protein sirtuin-3 (SIRT3) protected AML cells from cytarabine-induced apoptosis by inhibiting ROS production and by enhancing OXPHOS. Increased SIRT3 activity in AML cells resulted in resistance to chemotherapy (129). Together, these results showed that enhanced mitochondrial OXPHOS plays a major role in therapy resistance in AML.

Inhibition of BCL2 by venetoclax efficiently affected LSC survival by inhibition of OXPHOS and impairing energy homeostasis, resulting in upregulation of myeloid differentiation genes, and downregulation of cell cycle and proliferation genes (64) (**Table 1**). However, AML cells with high expression of the anti-apoptotic protein MCL1 showed resistance to BCL2 inhibitors (18, 64). To further reduce AML MRD load after venetoclax it may be an efficient strategy to use MCL1 inhibitors. Indeed, inhibition of MCL1 resulted in elimination of venetoclax-resistant AML (stem and progenitor) cells (18, 66–68) (**Table 1**). Several clinical trials are currently investigating the combination of BCL2 and MCL1 inhibitors, including AZD5991, AMG176 and MIK665 (68–70). In a clinical trial, treatment of elderly previously untreated AML patients with venetoclax in combination with the hypomethylating agents azacitidine or decitabine led to high overall response rates (13). This efficient response was due to targeting LSCs by inhibiting their amino acid uptake and catabolism, resulting in suppression of OXPHOS (65). Due to a more complex metabolic profile, LSCs derived from relapsed AML patients were less sensitive to this combination (130), highlighting again the importance of understanding the metabolic dynamics of LSCs during the course of disease.

In AML patients, the presence of mutations in IDH1 and IDH2 dysregulated mitochondrial function due to accumulation of 2-hydroxygluterate (2-HG). This resulted in increased ROS levels and activation of HIF-1*α* (131). LSCs residing within IDH1/2 mutated patients showed an increased dependency on BCL2, and were more sensitive to venetoclax, due to inhibition of cytochrome *c* oxidase by 2-HG (132). However, in a phase 2 clinical trial treating high-risk r/r AML patients with venetoclax, only 33% of the IDH1/2 mutated AML patients showed a CR. The effect of venetoclax treatment on the survival of LSCs was not determined in this trial (15).

The synthesis of HIF-1*α* in the hypoxic BM microenvironment induces upregulation of CXCR4 on the membrane of LSCs, thereby enhancing their migration ability, their anchorage in the BM niche and their resistance to therapy (7). Although targeting hypoxia and HIFs has been considered as potential therapeutic approaches for AML, several studies showed contrasting results (**Table 1**). CD34+CD38− LSCs have the highest levels of *HIF-1α*, and loss of HIF-1*α* led to the elimination of LSCs (71). In contrast to this result, loss of HIF-1*α* accelerated conversion of pre-leukemic cells to LSCs and shortened AML latency. Moreover, deletion of HIF-1*α* gave rise to faster progression of chemotherapy-treated MLL-AF9 AML cells (133).

Hypoxia-activating prodrugs (HAPs) are able to specifically target cells in hypoxic niches, as the active form of the drug is released under hypoxic conditions (134). The HAP TH-302 induced hypoxia-dependent apoptosis in AML cells, by reducing HIF-1*α* expression, thereby decreasing proliferation, inducing a cell-cycle arrest, and enhancing double-stranded DNA breaks (72). Administration of TH-302 after chemotherapy to mice with residual disease prolonged their survival (73), suggesting that specifically targeting of HIF-1*α* in the hypoxic niche may be a successful therapeutic strategy to specifically eliminate chemotherapy resistant AML (stem) cells.

## LSCs, the Bone Marrow Microenvironment and Therapy Resistance

There is emerging evidence that AML LSCs can remodel the BM niche into a leukemia-permissive microenvironment, thereby suppressing normal hematopoiesis. The complex interplay between LSCs and their microenvironment, including adhesion molecules, chemokines and cytokines, contribute to LSC survival, therapy resistance and disease relapse. Understanding these interactions is crucial for the development of effective drugs to overcome niche-mediated AML drug resistance.

Leukemia cells express the *β*-1 integrin receptor family members very late antigen-4 (VLA-4) and VLA-5, and the *β*-2 integrin LFA-1, which interact with the stromal ligands vascular cell adhesion molecule 1 (VCAM-1), fibronectin and intracellular adhesion molecule 1 (ICAM-1) to support attachment to the niche, thereby activating prosurvival and proliferative pathways in the leukemic blasts (135–137). Gene expression profiling of BM mesenchymal stromal cells (MSC) co-cultured with leukemia cells revealed upregulation of nuclear factor (NF)-*κ*B signaling, which reduced sensitivity to chemotherapy in the leukemia cells. Mechanistically, activation of the N*F*-κB signaling pathway was caused by the interaction of VLA-4 on the leukemia cells with ICAM-1 on the MSC (138).

HIF-1*α* signaling regulates LSC maintenance, quiescence and therapy sensitivity by promoting expression of VEGF, CXCR4, CXCL12 and SCF on both the AML blasts and the stromal cells (139). AML blasts and especially LSCs express CXCR4 on their surface and migrate in response to CXCL12 (140). The protective effects of the BM niche could be reduced by inhibition of the CXCL12–CXCR4 interaction (**Table 1**). For example, the CXCR4 antagonist plerixafor released HSCs and AML blasts from the BM niche, and the combination of plerixafor with cytarabine decreased tumor burden in an AML mouse model (74). Also in r/r AML patients, treatment with plerixafor enhanced the effect of chemotherapy (75). However, in newly diagnosed older AML patients, the clinical benefit of plerixafor was not shown, and mobilization of AML LSCs and progenitor cells after treatment was only observed in some patients (76). Targeting CXCR4 together with other membrane molecules involved in the attachment of AML (stem) cells to the BM niche may be an efficient strategy to release AML LSCs and enhance their sensitivity to therapy. Degradation of BET proteins with the BET proteolysis-targeting chimera ARV-825 resulted in downregulation of both CXCR4 and CD44 in AML cells, and as a result impairment of CXCL12-directed migration, increased oxidative stress, and downregulation of gene signatures associated with “stemness” and Wnt/*β*-catenin and Myc pathways. Importantly, treatment with ARV-825 alone and in combination with cytarabine decreased the number of LSCs (77).

Pro-inflammatory cytokines, such as tumor necrosis factor α (TNFα), interferon (IFN)-*α*, IFN-*β*, IFN-*γ*, IL-1 and IL-6, influence the adhesion of AML cells with their BM microenvironment and consequently AML survival and sensitivity to therapy, potentially through modulation of NF-*κ*B signaling. CD34+CD38- LSCs, but not normal HSCs or non-LSC AML blasts, showed constitutive NF-*κ*B activity due to autocrine TNF-α secretion, resulting in their expansion (141). Targeting transmembrane TNF-α increased sensitivity to chemotherapy, inhibited AML cell growth, and impaired AML engraftment in secondary serial transplantations (142). In addition to TNF-α, most AML cells express the pro-inflammatory cytokine IL-1, and especially IL-1β, enhancing the production of other pro-leukemic chemokines and thereby generating a pro-inflammatory niche. This pro-inflammatory environment promotes LSC and AML blast survival, proliferation and apoptosis-resistance (143, 144). Furthermore, the IL1R co-receptor IL1RAP is highly expressed on LSCs but not on HSCs of most AML patient samples, and involved in LSC self-renewal (32). Targeting IL1RAP could eliminate both leukemic bulk cells as well as LSCs and progenitor-enriched cell fractions of primary AML patient samples, while normal HSCs and progenitor cells were spared (145).

Several members of the transforming growth factor *β* (TGF*β*) family suppressed the growth of primary AML cells (144), and blockade of TGF*β* was therefore thought to enhance chemotherapy sensitivity of AML cells (**Table 1**). Indeed, in co-culture with human MSC, treatment of primary AML samples with a neutralizing TGF*β*1-antibody resulted in enhanced proliferation of both CD34+CD38− and CD34+CD38+ AML cell populations, and improved sensitivity to cytarabine (146). Blocking TGF*β* signaling using the neutralizing TGF*β* antibody 1D11 increased cytarabine-induced apoptosis of AML cells in hypoxic conditions. The combination of 1D11 with plerixafor and cytarabine decreased leukemia burden in a murine FLT3-mutated AML mouse model (78).

VEGF expressing endothelial cells in the BM niche protect VEGF receptor 3-expressing AML cells from chemotherapy-induced apoptosis, due to increased BCL2/Bax ratios (147). Moreover, treatment with a monoclonal VEGFC antibody reduced the clonogenic capacity of CD34-positive AML blasts, and induced their differentiation *via* the suppression of FOXO3A and inhibition of MAP/ERK (79) (**Table 1**). However, targeting VEGF signaling as novel therapeutic strategy has not been proven effective, as treatment with bevacizumab in AML patients showed controversial results in clinical trials. While bevacizumab after chemotherapy showed a favorable CR rate and duration in r/r AML patients that were resistant to the classical cytotoxic agents (80), in two other clinical trials with AML patients it showed not to be effective (81, 82).

## Adipocytes, AML LSCs and Therapy Resistance

In addition to glucose, proteins and fatty acids can also be metabolized to acetyl-CoA to drive the TCA cycle and OXPHOS in the production of ATP (148). As a result of stimuli from the BM microenvironment, such as hypoxia and nutrient availability, AML cells can modulate their metabolic state. Adipocytes, the major stromal cells present in the BM, support the survival and growth of AML cells by stimulating fatty acid oxidation (FAO) and mitochondrial OXPHOS as a result of fatty acid transfer (149). Moreover, adipocytes in the BM showed to impair the efficacy of chemotherapeutic drugs, and relapse rates after chemotherapy were much higher in mice that were obese than in mice with a normal body weight (149, 150). LSCs can induce lipolysis in adipocytes to induce FAO in AML (stem) cells by abundant fatty acids, thereby evading chemotherapy-driven elimination of the AML cells (**Table 1**). Only part of the LSC population in the BM express the fatty acid transporter CD36 (151), and these CD36+ LSCs showed to be highly proliferative and distinct from the pool of CD69 expressing LSCs that contain self-renewal potential (152). Further research should investigate if targeting the fatty acids uptake by CD36 would be a successful strategy to specifically eliminate AML LSCs. Furthermore, the lipid chaperone fatty acid binding protein 4 (FABP4) is involved in the interaction of adipocytes with leukemia cells, and its expression correlated with the activation of the peroxisome proliferator-activated receptor (PPAR) **γ** (153). FABP4 was increased in AML cells after culturing them with BM adipocytes (154), and downregulation of FABP4 resulted in increased survival of mice with Hoxa9/Meis1-driven murine leukemia (83).

BCL2 is directly reducing ROS generation (155), and BCL2 overexpression promoted the survival of low ROS-producing quiescent LSCs (64). Targeting FAO increased the production of ROS and caused apoptosis in AML cells (156). As targeting BCL2 by venetoclax eliminated low ROS-producing LSCs (64), targeting both FAO and BCL2 might be a successful synergistic approach to eliminate LSCs. FAO inhibition by etomoxir, an inhibitor of the FAO key rate-limiting enzyme carnitine O-palmitoyltransferase 1 (CPT1), disrupted metabolic homeostasis, increased ROS production, and subsequently induced expression of the integrated stress response (ISR) mediator activating transcription factor 4 (ATF4) in AML cells (84). Inhibition of CPT1 showed anti-AML effects (84, 85). CPT1 controls FAO by conjugating fatty acids with carnitine for translocation into the mitochondrial matrix. Expression of CPT1A is regulated by PPARs and the PPAR**γ** coactivator-1 (157), and inhibition of CPT1A by etomoxir not only directly eliminated leukemia cells but also sensitized them to cytarabine (85). Etomoxir induced an energetic shift towards low OXPHOS and resulted in increased anti-leukemia effects of cytarabine (36). Moreover, avocatin B, another inhibitor of FAO, was synergistic with cytarabine in inducing apoptosis in AML cells that were co-cultured with adipocytes by causing an increase in ROS production (84).

## Stress Response, LSCs and Therapy Resistance

Human HSCs are sensitive to environmental stress and prone to programmed cell death. HSCs ensure their persistence by using the ISR, also known as the unfolded protein response, in order to survive low-levels of stress caused by metabolic processes during normal homeostasis (158, 159). The ISR pathway balances the activation of apoptosis due to stress signals with survival pathways that protect the cell from dying (160). In response to stress stimuli, the eukaryotic translation initiation factor 2*α* (eIF2α) is phosphorylated by the stress-responsive eIF2*α* kinases general control non-derepressible 2 (GCN2), heme-regulated inhibitor, protein kinase R, and protein kinase RNA-like ER kinase (PERK). Phosphorylated eIF2*α* reduces global protein synthesis while allowing translation of specific genes, including ATF4, ATF5 and C/EBP Homologous Protein (161). Components of the eIF2*α* pathway, including GCN2 and ATF4, specifically contributed to survival of therapy-resistant cells during hypoxia. Activation of the ISR protects against ROS (162), and is therefore thought to be an important contributor to the survival of AML LSCs residing in hypoxic niches after therapy.

In response to ER stress human HSCs maintain their functionality preferentially through activation of the PERK-eIF2*α* IRS pathway (158). This pro-survival pathway was also shown to modulate the stress response of LSCs, thereby affecting their sensitivity to therapy and their survival (**Table 1**). In primary AML, CD34+CD38− LSCs contained lower eIF2*α* and elevated ATF4 levels as compared to the more differentiated CD34+CD38+ AML cell populations (159), highlighting that there is an increased activity of the ISR in LSCs, and implicating that ATF4 is a potential therapeutic target to eliminate LSCs. A mutation in FLT3, the FLT3-ITD, was shown to positively control ATF4 levels and enhanced autophagy in FLT3-ITD-mutated AML patient cells. Inhibition of ATF4 in FLT3-ITD-positive AML inhibited autophagy-dependent AML cell proliferation and tumor burden (**Table 1**). Moreover, inhibition of autophagy by VPS34 inhibitors abolished resistance to FLT3 inhibitors in murine xenograft models (86). The antiparasitic drug atovaquone enhanced the phosphorylation of eIF2α, increased ATF4 protein levels and transcription of ATF4 target genes, and inhibited mitochondrial OXPHOS, which resulted in growth arrest and apoptosis of AML patient cells (87). Moreover, activation of the PERK signaling pathway and subsequent activation of autophagy induced resistance to G9a inhibition in AML LSCs. Combination treatment of PERK and G9a inhibitors induced apoptosis in LSCs (88). Together, these studies suggest that therapeutic targeting of the PERK-eIF2*α*-ATF4 ISR pathway may be an efficient approach to eradicate AML LSCs.

## Discussion and Conclusion

One of the biggest challenges in treating AML is the development of relapse after initial treatment. Even with high remission rates, therapy resistance and relapse are often appearing and are the major obstacles to a cure. AML “persisters” (MRD) after initial therapy are caused by various mechanisms that co-exist, including epigenetic, transcriptional, and metabolic processes. Successful therapeutic strategies targeting AML MRD and LSCs will increase efficacy of treatment and finally the survival of patients. Increasing the knowledge on the mechanisms driving this persistence, and also on the changes in identity of MRD and LSCs during the course of the disease is crucial for the development of successful therapeutic strategies to overcome therapy resistance and to inhibit leukemia-initiating potential. Similarities and differences between normal HSCs and AML LSCs and studying LSC heterogeneity and intra- and interpatient plasticity are key for therapy development specifically eradicating LSCs at any time during the disease course. Persistent LSCs are characterized by their quiescent state and might exist prior to drug treatment; however, they might also become resistant upon exposure to therapy. At relapse, there are substantially more LSCs and phenotype diversities of LSCs than at diagnosis, indicating that the current therapeutic treatments induce dramatic changes in the LSC and relapse-initiating cell compartment. This indicates that there is a high degree of phenotypic plasticity to impose “stemness” on leukemia cells and shows the importance of studying leukemia re-initiating cells after the initial therapy. Future characterization of leukemia blasts and LSCs should therefore be performed after treatment to identify specific leukemia-relapse initiating markers that could be used not only to prognostically detect MRD, but also to early detect relapse and to be used as therapeutic targets delaying or even preventing relapse.

There is a complex interplay between AML (stem) cells, their BM niche, and the outcome of treatment. This interplay is significantly influenced by signaling events from the BM niche affecting metabolism, epigenetic processes, stress responses and transcriptomes of the AML cells, and all subsequently affecting the level of leukemia (stem) cell death induced by therapy. Therefore, targeting the BM microenvironment by novel therapeutic strategies will also be crucial to overcome AML drug resistance. For example, targeting the energy consumption of LSCs in combination with inhibition of BCL2 can potentially eradicate residual chemotherapy-resistant LSC populations. Both FAO and BCL2 directly reduce ROS generation, promoting the survival of low ROS-producing quiescent LSCs. The combination of venetoclax and a FAO inhibitor might be a successful approach to eliminate LSCs with adapted energy homeostasis. Furthermore, the differentiation stage of the AML cells plays an important role in the response to therapy. AML cases originating from HSCs or MPPs, thus immature AML cases with high EVI-1 expression, may be successfully treated with venetoclax, while more differentiated AML cases may be successfully treated with alternative therapies that are more efficient in the eradication of differentiated AML cells.

In conclusion, characterization of AML (stem) cells at the single cell level during the course of the disease and especially at MRD will provide valuable insights into the mechanisms of AML persistence and relapse-initiation and is key for development of successful treatment strategies that reduce or prevent relapse.

## Author Contributions

NV, FD, and LS wrote the manuscript. All authors contributed to the article and approved the submitted version.

## Funding

This study was supported by the Dutch Cancer Society grant 12805.

## Conflict of Interest

The authors declare that the research was conducted in the absence of any commercial or financial relationships that could be construed as a potential conflict of interest.
